# Automated segmentation of retinal vessel using HarDNet fully convolutional networks

**DOI:** 10.1371/journal.pone.0330641

**Published:** 2025-09-08

**Authors:** Yuanpei Zhu, Yong Liu, Xuezhi Zhou

**Affiliations:** 1 School of Physics and Electronic Engineering, Xinxiang University, Xinxiang, China; 2 School of Information Science and Engineering, Wuhan University of Science and Technology, Wuhan, China; 3 School of Medical Engineering, Xinxiang Medical University, Xinxiang, China; Khalifa University, UNITED ARAB EMIRATES

## Abstract

Computer-aided diagnostic (CAD) systems for color fundus images play a critical role in the early detection of fundus diseases, including diabetes, hypertension, and cerebrovascular disorders. Although deep learning has substantially advanced automatic segmentation techniques in this field, several challenges persist, such as limited labeled datasets, significant structural variations in blood vessels, and persistent dataset discrepancies, which continue to hinder progress. These challenges lead to inconsistent segmentation performance, particularly for small vessels and branch regions. To address these limitations, we propose an enhanced HarDNet-based model that integrates HarDNet modules, Receptive Field Block (RFB) modules (designed to capture multi-scale contextual information), and Dense Aggregation modules. This innovative architecture enables the network to effectively extract multi-scale features and improve segmentation accuracy, especially for small and complex structures. The proposed model achieves superior performance in retinal vessel segmentation tasks, with accuracies of 0.9685 (±0.0035) on the DRIVE dataset and 0.9744 (±0.0029) on the CHASE_DB1 dataset, surpassing state-of-the-art models such as U-Net, ResU-Net, and R2U-Net. Notably, the model demonstrates exceptional capability in segmenting tiny vessels and branch regions, producing results that closely align with the gold standard. This highlights its significant advantage in handling intricate vascular structures. The robust and accurate performance of the proposed model underscores its effectiveness and reliability in medical image analysis, providing valuable technical support for related research and applications.

## Introduction

The convergence of artificial intelligence (AI) and medical imaging has transformed diagnostic methodologies, particularly in retinal vessel segmentation, which has become indispensable in modern healthcare. This technology offers significant potential for advancing clinical applications by enabling early detection of systemic conditions such as diabetic retinopathy, hypertension, and various ocular pathologies through the analysis of retinal vascular morphology [1]. AI-assisted systems further provide ophthalmologists with enhanced decision-making tools, improving diagnostic accuracy and therapeutic outcomes [2].

The rapid proliferation of ophthalmic imaging data, driven by advances in optical coherence tomography (OCT) and the growing emphasis on early disease detection, has rendered manual analysis impractical. The analysis of the DRIVE and CHASE_DB1 datasets highlights critical factors affecting imaging quality. High-precision systems, with resolutions of 5–10 µm and signal-to-noise ratios (SNR) of 30–40 dB, outperform conventional systems (20–50 µm resolution, 10–20 dB SNR). Operator expertise also plays a pivotal role, with experienced practitioners maintaining image quality above 95%, while novices often experience a 10–20% decline. Empirical evidence shows that a 10% reduction in resolution decreases segmentation accuracy by 5–10%, while a 5 dB increase in noise reduces accuracy by 3–5%. Structural variations in vessel morphology can lead to segmentation fluctuations of 10–15%.

Automated segmentation algorithms have emerged as essential tools, addressing these challenges by enhancing efficiency, accuracy, and resource utilization. These methods underpin advanced applications such as multimodal image registration, arteriovenous differentiation, and biometric identification.

Convolutional neural networks (CNNs) have become the dominant paradigm in medical image segmentation due to advancements in computational infrastructure. Despite significant progress in retinal vessel analysis, challenges persist in segmenting microvascular structures. CNNs often struggle to capture fine morphological details and complex branching patterns, which are critical for accurate disease diagnosis [3].

Dataset limitations further complicate these challenges. Most publicly available datasets contain only tens to hundreds of annotated images, insufficient for training robust models. The U-Net architecture, introduced by Ronneberger et al. [4], addressed this by integrating multi-scale features through skip connections, achieving improved performance with limited data. However, existing U-Net variants still face issues with cross-dataset generalization and sensitivity to microvascular structures, particularly at bifurcation points and crossings.

To overcome these limitations, we propose HarDNet (Harmonic Densely Connected Network), an advanced architecture designed for retinal vessel segmentation in fundus images. HarDNet incorporates three key components: the HarDNet module, the Receptive Field Block (RFB) module, and the Dense Aggregation module, addressing challenges in micro-vessel segmentation, multi-scale feature extraction, and dataset generalization. Experimental evaluations on the DRIVE and CHASE_DB1 datasets demonstrate superior performance in segmentation accuracy, particularly in micro-vessel detection and robustness across datasets [5].

## Related work

Retinal vessel segmentation methods can be categorized into unsupervised and supervised approaches based on their requirement for manual data labeling. Unsupervised techniques, including multi-scale vascular enhancement, multi-threshold detection, matched filtering, morphological transformation, and model-based algorithms, primarily depend on handcrafted features and heuristic rules [6]. Although computationally efficient, these methods often exhibit limitations in handling complex vascular structures and image noise, resulting in constrained accuracy and generalizability. In contrast, supervised methods leverage labeled datasets to train segmentation models, with convolutional neural network (CNN)-based approaches gaining significant attention due to their capacity for automatic feature learning, local perception, parameter sharing, and pooling operations, which collectively reduce parameter dimensionality and enhance training efficiency. Nevertheless, CNN-based methods face challenges including computational complexity, limited cross-dataset generalization, and difficulties in preserving fine-grained details crucial for medical image analysis.

The U-Net architecture, a cornerstone in medical image segmentation, employs a unique three-component structure comprising down-sampling, feature fusion, and up-sampling paths [6]. The down-sampling pathway progressively reduces spatial dimensions while extracting hierarchical features through convolutional operations. Feature integration is achieved through concatenation of corresponding layers from the down-sampling and up-sampling paths, while the up-sampling pathway reconstructs spatial resolution through transposed convolutions. Skip connections between layers facilitate multi-scale feature aggregation and depth supervision, enabling enhanced edge recovery and detailed segmentation. However, the fixed encoder-decoder architecture of U-Net presents limitations in handling scale variations and complex textures, particularly in imbalanced medical datasets.

Recent advancements in segmentation networks have demonstrated substantial progress through architectural innovations and module integration. Enhanced variants such as ResUNet [7], ResUNet++ [8], and DoubleU-Net [9] have incorporated advanced CNN backbones and supplementary modules including spatial pyramid pooling [10] and attention mechanisms [11]. These improvements have expanded receptive fields and enhanced multi-scale information integration. For instance, DoubleU-Net integrates Atrous Spatial Pyramid Pooling (ASPP) [12] between encoder and decoder stages to address scale variation, while PraNet [13] incorporates the Receptive Field Block (RFB) [14] module in skip connections to capture comprehensive multi-scale visual information. The integration of attention mechanisms in models like PraNet, PolypSeg [15], and ABC-Net [16] has further advanced pixel-level semantic segmentation in medical imaging.

Despite these advancements, enhanced U-Net variants face computational challenges. ResUNet [7] and ResUNet++ [8] employ deeper CNN architectures that improve feature extraction at the cost of increased computational complexity and hardware requirements. Similarly, the incorporation of ASPP in DoubleU-Net [9] and the RFB module in PraNet [13], while effective for multi-scale feature extraction, leads to a significant increase in computational complexity. This performance-efficiency trade-off presents practical limitations in resource-constrained medical applications.

Emerging architectures have introduced novel approaches to address these limitations. TransUNet integrates transformer-based global context modeling with the localization capabilities of U-Net, while UNet3 + implements full-scale skip connections to enhance multi-scale feature aggregation. Attention U-Net integrates attention gates to emphasize salient regions, demonstrating superior performance in complex structure segmentation and imbalanced datasets. However, these advanced architectures often incur additional computational costs, potentially limiting their clinical applicability.

HarDNet [17] presents a computationally efficient alternative through its innovative use of depthwise separable convolutions, achieving state-of-the-art performance on DRIVE and CHASE DB1 datasets while maintaining efficient inference speeds. The architecture exhibits high parameter efficiency and superior accuracy, rendering it particularly well-suited for medical imaging tasks, where precise and reliable performance is of critical importance. However, its reliance on depthwise convolutions may constrain the capture of complex spatial dependencies in textured regions. Current research directions focus on integrating supplementary modules such as ASPP [12] and attention mechanisms [11] to enhance performance without substantially increasing computational requirements, potentially offering a balanced solution for clinical implementation.

## Proposed harmonic DenseNet

**Fig 1:**
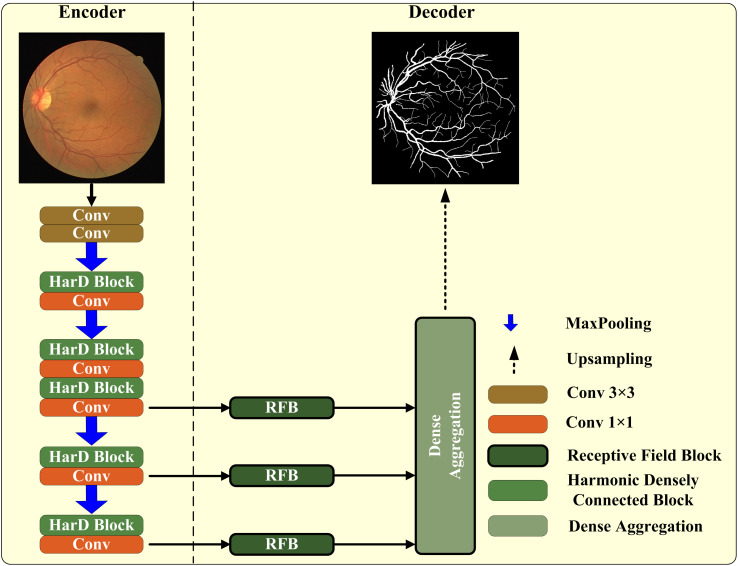
The overview of the proposed HarDenseNet. HarDNet Block was used to replace the original convolution operation in the down-sampling process, RFB(Receptive Field Block Module) block was used for skip connections, and Dense Aggregation was used in the up-sampling process.

The structure of the entire network is shown in Fig 1. Inspired by the U-Net model, we modified the original framework accordingly. HarDNet Block are used to replace the original convolution operation in the down-sampling process, RFB(Receptive Field Block Module) block are used for skip connections, and Dense Aggregation are used in the up-sampling process.

### HarDNet block

**Fig 2:**
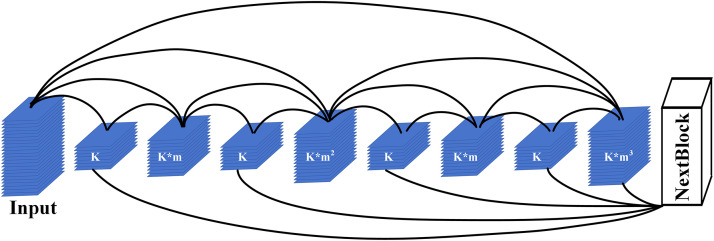
HarDNet block overview.

Fig 1 illustrates the architecture of the proposed HarDNet, which is built upon a U-Net-like encoder-decoder structure. The encoder backbone is based on HarDNet, an improved version of the original DenseNet [18], as detailed in Fig 2. HarDNet optimizes the dense block design by reducing the number of shortcuts to minimize memory traffic and improve inference speed, while simultaneously increasing the channel width of key layers to compensate for potential accuracy loss. Additionally, it incorporates a small number of Conv1x1 layers to enhance computational density. These modifications to the encoder structure align with the U-Net framework by maintaining its core design principles-such as skip connections and feature aggregation-while addressing specific limitations, such as memory efficiency and computational cost, which are critical for medical image segmentation tasks like retinal vessel segmentation. This ensures that the network retains the ability to capture fine-grained details and contextual information, which are essential for accurate segmentation.

By improving HarDNet, it is not only able to reduce inference time compared to DenseNet and ResNet [19], but also has a higher accuracy. Some other experiments also show that the image segmentation of FC-HarDNet70 on the urban landscape dataset [20] has also reached the most advanced level. Therefore, we decided to use HarDNet68 as the model backbone for semantic segmentation of retinal blood vessel images.

### Cascaded partial decoder

The outstanding performance of U-Net in the medical field has made the framework of U-Net used in the later medical image segmentation methods, or improved on the basis of U-Net. the proposed framework also takes into account the advantages of the U-Net structure. However, in subsequent experiments, we considered issues such as inference time and accuracy, and ended up not using HarDBlock (HarDBlk) in the Decoder part, unlike FCHarDNet.

The related research of the cascade partial decoder [21] found that the shallow layer features have the characteristics of high resolution and occupy computing resources, and the deep layer information can also better represent the spatial details of the shallow layer information. We try to give up shallow features and do more calculations on deeper features. At the same time, by adding appropriate convolution and skip connection in the network structure, the aggregation of different scale feature graphs is realized.

### Receptive field block module

**Fig 3:**
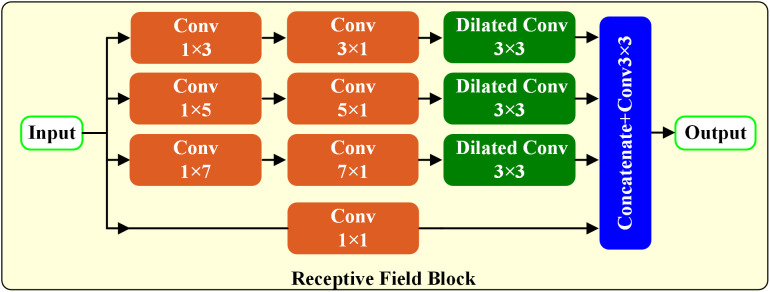
Receptive field block overview.

Fig 3 shows the receptive field block [22]. It reinforces the deep features learned from the lightweight CNN backbone. By using multi-branch convolution and empty convolution layers with convolution kernels of different sizes, features with different acceptance domains can be generated. These features are then combined and the final representation generated by concatenation operations and convolution kernels into 3x3 convolution.

We incorporate this module into the skip connection as suggested by [21], thereby expanding the proposed receptive fields from feature maps of varying resolutions.

### Dense aggregation

**Fig 4:**
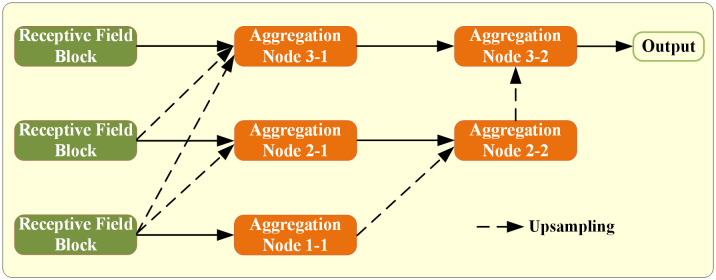
Aggregation module overview.

We conduct dense aggregation through element-wise multiplication, as depicted in Fig 4. Specifically, feature maps from different layers are first up-sampled to a uniform spatial resolution using bilinear interpolation, ensuring precise alignment across scales. Following this, the up-sampled feature maps are combined with corresponding skip connection feature maps via element-wise multiplication. This multiplicative aggregation mechanism enhances the capacity of the model to capture fine-grained details and spatial dependencies, which is particularly critical for tasks such as retinal vessel segmentation where accurate localization of thin structures is essential. The use of element-wise multiplication, as opposed to simple concatenation or addition, facilitates a more selective integration of features by emphasizing regions of high activation while suppressing irrelevant background information. This approach draws inspiration from the success of attention mechanisms in computer vision, where multiplicative interactions have been demonstrated to effectively highlight salient features. Moreover, the dense aggregation strategy ensures that multi-scale information is preserved and efficiently utilized throughout the network, thereby enhancing both the robustness and accuracy of the segmentation results.

Let the input to the Dense Aggregation Block be a set of feature maps from previous layers, denoted as:


$$\left\{X_{1},X_{2},\cdots ,X_{n}\right\}$$ (1)

Where $X_{i}\in {\mathbb{R}}^{H\times W\times C_{i}}$ represents the *i*-th feature map with heigh *H*, width *W*, and *C*_*i*_ channels.

The the Dense Aggregation Block aggregates features from multiple layers through concatenation. The concatenated feature map *F*_*cat*_ is given by:


$$F_{cat}=Concat\left(X_{1},X_{2},\cdots ,X_{n}\right)$$ (2)

Where $F_{cat}\in {\text{R}}^{H\times W\times \sum_{i=\text{1}}^{n}C_{i}}$.

The concatenated features are passed through a transformation fuction to reduce dimensionality and extract meaningful information. This can expressed as:


$$F_{trans}=\sigma \left(W*F_{cat}+b\right)$$ (3)

where:

*W* is the weight matrix of the convolutional layer,

*b* is the bias term,

* denotes the convolution operation,

σ is a non-linear activation fuction

$F_{trans}\in {\mathbb{R}}^{H\times W\times C_{i}}$ is the transformed feature map with $C^{\prime }$ output channels.

The Dense aggregation Block Combines the transformed features with the original input features through element-wise addition or concatenation. For addition:


$$F_{out}=F_{trans}+X_{skip}$$ (4)

Where $F_{out}\in {\text{R}}^{H\times W\times C^{\prime \prime }}$, $C^{\prime \prime }=C^{\prime }+C_{skip}$

### Loss function

In the realm of object detection tasks, the IoU (Intersection over Union) loss function is pivotal in optimizing the localization accuracy of the model.The IoU metric is widely used to quantify the degree of overlap between predicted and ground truth bounding boxes. However, it has limitations in capturing differences in distance, shape, and aspect ratio between the two boxes. Specifically, IoU only evaluates the overlapping area and does not account for non-overlapping regions or geometric discrepancies. In scenarios where there is no overlap between the predicted and ground truth bounding boxes, the IoU value becomes zero. This can lead to challenges during the training process, particularly with respect to gradient vanishing, as the lack of overlap provides no meaningful feedback for model optimization. BCELoss effectively quantifies the discrepancy between the predicted probability distribution and the actual labels, thereby guiding the optimization process of the model. In binary classification tasks, BCELoss evaluates the prediction errors for both positive and negative samples, ensuring that the model considers the classification accuracy of both classes during training. However, BCELoss exhibits high sensitivity to probability predictions, particularly when probabilities are near 0 or 1, where minor changes can result in significant fluctuations in the loss value. This sensitivity is especially problematic in imbalanced datasets, as it may lead the model to favor predicting the majority class. The strategic amalgamation of these two loss functions significantly augments the overall performance of the model in object detection tasks, underscoring their indispensable role in advancing the field. We can express it as $L=\lambda_{1}L_{IoU}^{w}+\lambda_{2}L_{BCE}^{w}$, both $\lambda_{1}$ and $\lambda_{2}$ take 0.5. Generally, the IoU is calculated as


$$L_{IoU}=1-\frac{\sum_{r=1}^{H}\sum_{c=1}^{W}S(r,c)G(r,c)}{\sum_{r=1}^{H}\sum_{c=1}^{W}\left[S(r,c)+G(r,c)-S(r,c)G(r,c)\right]}$$ (5)

In the case of binary classification, the BCE formula of binary cross entropy is


$$l_{BCE}=-\sum_{\left(r,c\right)}\left[G(r,c)\text{lg}\left(S(r,c)\right)+\left(1-G(r,c)\right)\text{log}\left(1-S(r,c)\right)\right]$$ (6)

where r and c denote the coordinates of the pixel points, while H and W represent the height and width of the input image, G(r, c)∈{0,1} is the ground truth label of the pixel point (r, c), S(r, c) is the prediction probability for which it is the target significance category. IoU quantifies the overlap ratio between predicted and ground truth regions, while BCE measures the classification error. To enhance the focus of the model on object edges in image processing, a weighted loss calculation method is employed. Specifically, a 31 × 31 matrix is constructed by extracting pixel values from a 15-pixel neighborhood in four cardinal directions (up, down, left, and right) surrounding each target pixel. The mean value of this matrix is subsequently computed. The absolute difference between this mean value and the target pixel value is then determined, representing the local variation. This difference is weighted proportionally to its magnitude in the final loss computation. This methodological approach directs the attention of the model to image edges during training. The process exclusively considers the absolute differences between each pixel and its local neighborhood, as the computational outcome depends solely on the magnitude of these differences.

## Experiments

### Datasets

The proposed HarDNet was evaluated using the DRIVE and CHASE_DB1 retinal image datasets. These datasets are widely recognized in the field of retinal vessel segmentation due to their reliable ground truth annotations and diverse characteristics. The original structure of the DRIVE dataset, in particular, significantly influences the segmentation process. DRIVE contains 40 color fundus images, which are divided into 20 for training. The ratio of those used for training and validation is 9:1, and there are 20 test images [22]. This predefined split facilitates consistent benchmarking of segmentation algorithms, making it an appropriate choice for assessing the generalization capability of the proposed model. Nevertheless, the limited size of the training dataset requires the application of data augmentation techniques to improve model performance. Moreover, the dataset comprises manually curated annotations developed by human experts, which serve as the reference standard for both training and evaluation. These annotations are critical to supervised learning; however, they may introduce inconsistencies due to variations in human judgment, which could potentially affect the accuracy of segmentation.

The images of the DRIVE dataset have a resolution of 565 × 584 pixels, which is sufficient for capturing fine details like small blood vessels but requires careful preprocessing to handle noise and variability in lighting and contrast. The dataset also includes field-of-view (FOV) masks, which are essential for focusing the segmentation on the relevant retinal region and excluding the background. Proper utilization of these masks during preprocessing is crucial for accurate segmentation.

CHASE_DB1, containing 28 retinal fundus images, complements DRIVE by offering a slightly different set of images with a resolution of 999 × 960 pixels. The first 20 images are typically used for training, and the remaining 8 for testing [23]. This dataset provides diversity in retinal structures and vessel patterns, helping to test the robustness of segmentation models across varied imaging conditions. Like DRIVE, CHASE_DB1 also provides manually segmented binary vessel maps as ground truth, which are used for performance comparison.

To adapt to the proposed architecture, we resized each image in both datasets to 592 × 592 and 1008 × 1008. However, during the evaluation phase, the segmentation results were cropped back to their original resolutions to ensure accurate performance measurements. The combination of these datasets allows us to evaluate the ability of the proposed model to generalize across different resolutions, imaging conditions, and vessel structures, while the original structure of DRIVE, including its predefined splits, annotations, and FOV masks, plays a critical role in shaping the segmentation process. To minimize discrepancies among the data, it is necessary to implement preprocessing measures including image standardization, grayscale normalization, noise reduction, contrast enhancement, and data augmentation. These steps are essential for improving the accuracy and robustness of the segmentation model.

### Implementation details

The experiments were conducted on a desktop computer equipped with an Intel Core i5-9400 processor, 32GB of RAM, and an NVIDIA 2080Ti GPU featuring 11GB of memory. To optimize model parameters, we utilized the Adam optimization algorithm. To accelerate network convergence, we implemented a phased training approach by dynamically adjusting parameter updates during the training process [24]. In experiments carried out on the DRIVE dataset, we established an initial learning rate of 0.003 for the first 150 epochs, which was then reduced to 0.0001, achieving satisfactory outcomes. Furthermore, the hyperparameters were set to 0.9 and 0.999 respectively. This approach is also applicable to the CHASE DB1 dataset. During the preprocessing stage of image data, pixel values are normalized to a fixed range ([0, 1]) to facilitate more efficient model convergence. Histogram equalization is applied to enhance image contrast and delineate vascular structures with greater clarity. To optimize network performance, a variety of data augmentation techniques have been implemented. For each image input, one of the following methods is randomly selected for augmentation: horizontal flipping, vertical flipping, contrast adjustment, or the addition of Gaussian noise. The experiment employs a manual stopping mechanism. The weights are saved every 20 iterations, and then loaded to carry out experiments on the test set to acquire the performance of the model.

### Evaluation metrics

Quantitative evaluation permits a more comprehensive and insightful assessment of the model. Through systematic pixel-wise comparison between the segmentation outcomes and the corresponding ground truth, the results were categorized into four distinct classes: true positive (TP), false positive (FP), false negative (FN), and true negative (TN). To conduct a comprehensive and rigorous assessment the performance of the model, key performance indicators were employed for evaluation, specifically including sensitivity (Se), specificity (Sp), F1 score (F1), and accuracy (ACC).


$$S\text{e}=\frac{TP}{TP+FN},$$ (7)

$$Sp=\frac{TN}{TN+FP},$$ (8)

$${\text{precision}}_{K}=\frac{TP}{TP+FP},$$ (9)

$$F1={\left(\frac{1}{\text{n}}\sum \frac{2\cdot {\text{precision}}_{K}\cdot {\text{recall}}_{K}}{{\text{precision}}_{K}+{\text{recall}}_{K}}\right)}^{2},$$ (10)

$$A\text{cc}=\frac{TP+TN}{TP+FN+TN+FP}.$$ (11)

Sensitivity is a pivotal metric for evaluating the capability of the model to accurately detect positive instances (vessels). A high sensitivity ensures that the model captures the majority of vessel pixels, thereby significantly reducing the probability of false negatives. This attribute is particularly crucial for the early detection and diagnosis of pathological conditions, such as diabetic retinopathy, where missing vascular abnormalities could lead to severe clinical consequences. Conversely, specificity plays a fundamental role in assessing the ability of the model to correctly identify negative instances (background). A high specificity indicates that the model can effectively discriminate between background and vessel pixels, thereby minimizing the occurrence of false positives and improving the overall reliability of the segmentation results. The Area Under the Curve (AUC) serves as a holistic performance metric, encapsulating the classification efficacy of the model across a range of decision thresholds. An AUC value approaching 1 denotes exceptional classification performance, offering a robust measure of the balanced accuracy, particularly in contexts characterized by significant class imbalance of the model.

## Results

**Fig 5:**
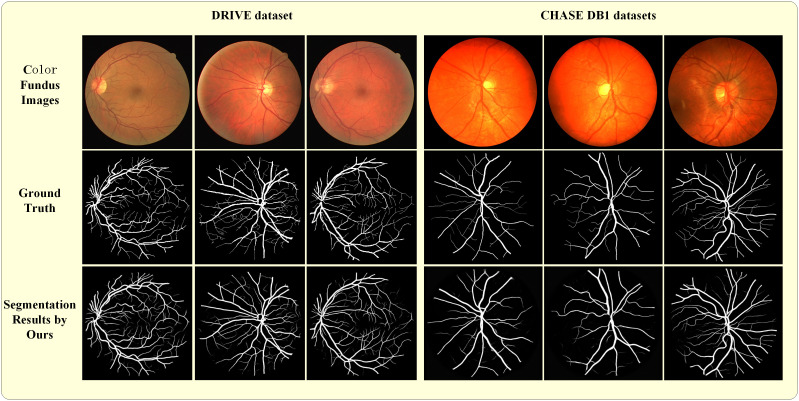
A demonstration of some examples from the DRIVE and CHASE DB1 datasets experiment. The three columns on the left are from the DRIVE dataset and the three columns on the right are from the CHASE DB1 datasets. The first line are the color fundus images from the two data sets, the second line is the corresponding ground truth, and the third line is the segmentation results based on the proposed method.

Fig 5 shows the partial segmentation results of the proposed method in DRIVE and CHASE DB1 datasets. When comparing the proposed segmentation outcomes with the annotations of the two datasets, the results of the proposed model demonstrate a close approximation to the gold standard, particularly in the region of small vascular branches. Furthermore, an analysis was conducted to evaluate the impact of different blocks on the results.

**Fig 6:**
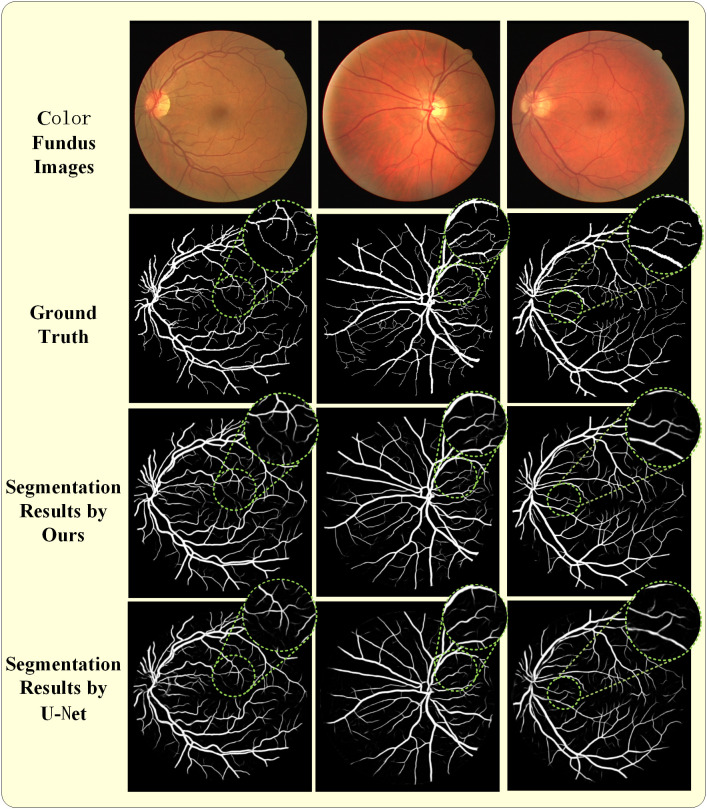
Comparison experiment between the proposed method and U-Net on DRIVE dataset, and the corresponding area is enlarged: The first line are the color fundus image from the Drive dataset; The second line are the corresponding manual annotations; The third line are the proposed segmentation results; And the fourth line are the U-Net segmentation results.

**Fig 7:**
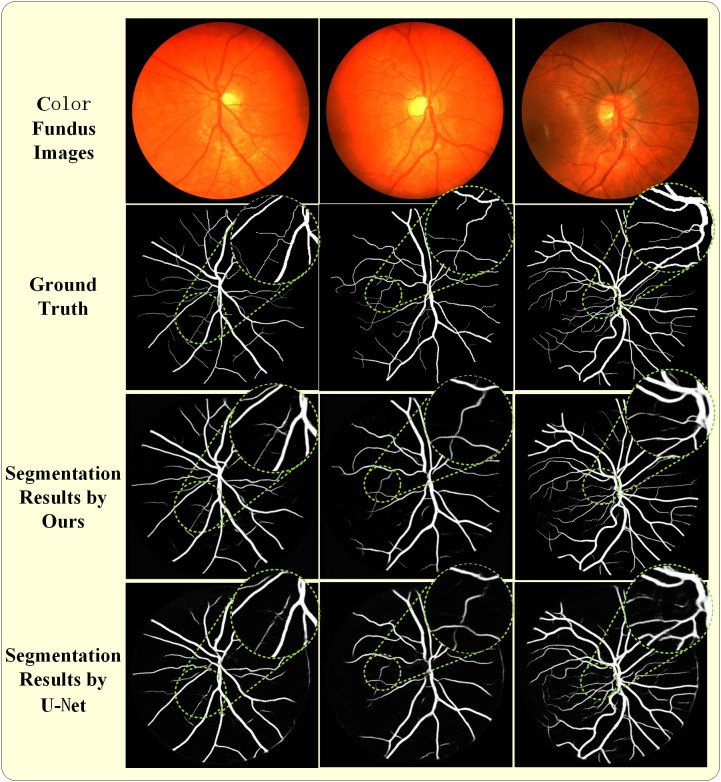
Comparison of the proposed HarDNet and U-Net on CHASE DB1 dataset, and the corresponding area is enlarged: First line are color fundus images; The second line are ground truth; The third line are segmentation result by The third line is; The last line are segmentation result by U-Net.

A comprehensive evaluation of the proposed method was performed by comparing its segmentation performance with that of the widely adopted U-Net model in medical image segmentation. Representative results are shown in Figs 6 and 7. Fig 6 illustrates an example from the DRIVE dataset: the first row shows the fundus color image; the second row displays the manually annotated ground truth; the third row presents the output from the proposed method; and the fourth row shows the segmentation result from U-Net. Fig 7 provides a comparative analysis of segmentation outcomes on the CHASE DB1 dataset. To emphasize the differences between the proposed method and U-Net, the region of interest was magnified, as shown by the green circle. Based on these findings, it can be inferred that the proposed approach successfully delineates finer vascular structures, particularly capillaries, with enhanced precision.

Figs 6 and 7 display the segmentation results from the Drive and Chase datasets, with selected regions zoomed-in to emphasize fine details. Each figure is organized into four rows: the first row shows the original images, the second row presents the ground truth, the third row showcases the segmentation results of the proposed model, and the fourth row compares these results with those from the U-Net model. A detailed comparison reveals that the proposed model demonstrates superior alignment with the ground truth compared to the U-Net model. This performance highlights the capability of the model to achieve state-of-the-art accuracy, underscoring the substantial potential for clinical applications in retinal imaging.

Finally, the improved HarDNet model was compared with several state-of-the-art methods on two publicly available datasets, DRIVE and CHASE DB1, and their results are presented in Table 1. To objectively evaluate the segmentation results, a variety of metrics are employed, including sensitivity (Se), specificity (Sp), accuracy (ACC), F1 score, and area under the curve (AUC). Among these evaluation criteria, the proposed HarDNet model demonstrates superior performance across both the DRIVE and CHASE DB1 datasets. Specifically, the proposed model achieves the highest AUC values (exceeding the top-performing model by 0.21% and 0.05%, respectively), the highest accuracy rates (outperforming the best model by 1.07% and 0.83%), and the highest sensitivity scores. Additionally, the F1 score and specificity remain highly competitive. Consequently, the proposed method establishes state-of-the-art performance in retinal vessel segmentation.

**Table 1:** Results of different methods on DRIVE and CHASE DB1.

Methods	DRIVE					CHASE DB1				
	Se	Sp	F1	ACC	AUC	Se	Sp	F1	ACC	AUC
U-Net [25]	0.7537	0.9820	0.8142	0.9531	0.9755	**0.8288**	0.8288	0.7783	0.9578	0.9578
ResU-Net [26]	0.7726	0.7726	0.8149	0.9553	0.9553	0.7726	0.7726	0.7800	0.9553	0.9553
Orlando et. al. [27]	0.7897	0.9684	0.7857	0.9454	0.9506	0.7277	0.9712	0.7332	0.9458	0.9524
Yan et al. [28]	0.7653	0.9818	–	0.9542	0.9752	0.7633	0.9809	–	0.9610	0.9781
R2U-Net [29]	0.7799	0.9813	0.8171	0.9556	0.9784	0.7756	0.7756	0.7928	0.9634	0.9634
LadderNet [30]	0.7856	0.9810	0.8202	0.9561	0.9793	0.7978	0.9818	0.8031	0.9656	0.9839
RU-Net [31]	0.7751	0.7816	0.8155	0.9556	0.9782	0.7459	0.9836	0.7810	0.9622	0.9803
DEU-Net [32]	0.7940	0.9816	**0.8270**	0.9567	0.9772	0.8074	0.9821	**0.8037**	0.9661	0.9860
Vessel-Net [33]	0.8038	0.9802	**–**	0.9578	0.9821	0.8132	0.9814	**–**	0.9661	0.9860
Dense-dilate Unet [34]	0.8126	0.9788	**–**	0.9594	0.9796	0.8268	0.9773	**–**	0.9637	0.9812
Ours	**0.8042**	**0.9843**	0.8178	**0.9685**	**0.9842**	0.8099	**0.9857**	0.8002	**0.9744**	**0.9865**

Table 2 demonstrates the significance comparison between the traditional U-Net and RES-UNet models on the DRIVE and CHASE datasets. In this table, the performance of the proposed model is used as the benchmark for P-value comparison and use multi-model ensemble verification.

**Table 2:** P-values for performance differences between the proposed method and baselines.

Methods	DRIVE	CHASE DB1
	P-value vs Baseline	
U-Net	0.0025	0.0018
ResU-Net	0.0034	0.0043
Ours	–	–

Furthermore, the integration of Receptive Field Block (RFB) modules and Dense Aggregation (DA) blocks into the model architecture can significantly enhance network performance. Additionally, innovative supervision mechanisms enable more effective monitoring the learning process of the network. On the DRIVE dataset, substantial improvements were observed in Sensitivity (Se), Accuracy (Acc), and Area Under Curve (AUC), achieving respective values of 0.8042, 0.9685, and 0.9842. Similarly, for the CHASE dataset, notable advancements were achieved in Specificity (Sp), Accuracy (Acc), and AUC, reaching values of 0.9857, 0.9744, and 0.9865, respectively.

**Fig 8:**
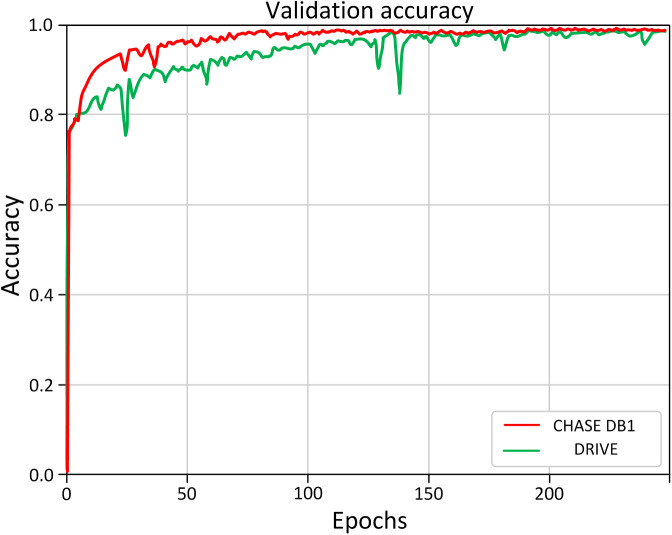
The validation accuracy curves obtained during the training processes of the two datasets, DRIVE and CHASE DB1.

**Fig 9:**
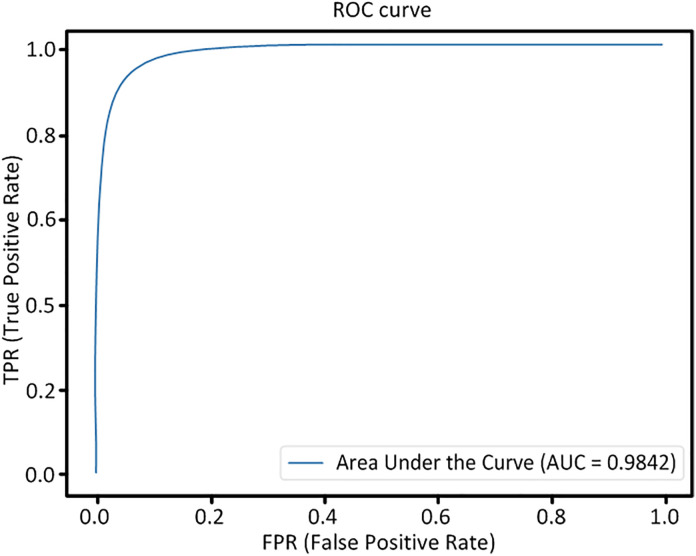
The ROC curve obtained from the DRIVE dataset.

**Fig 10:**
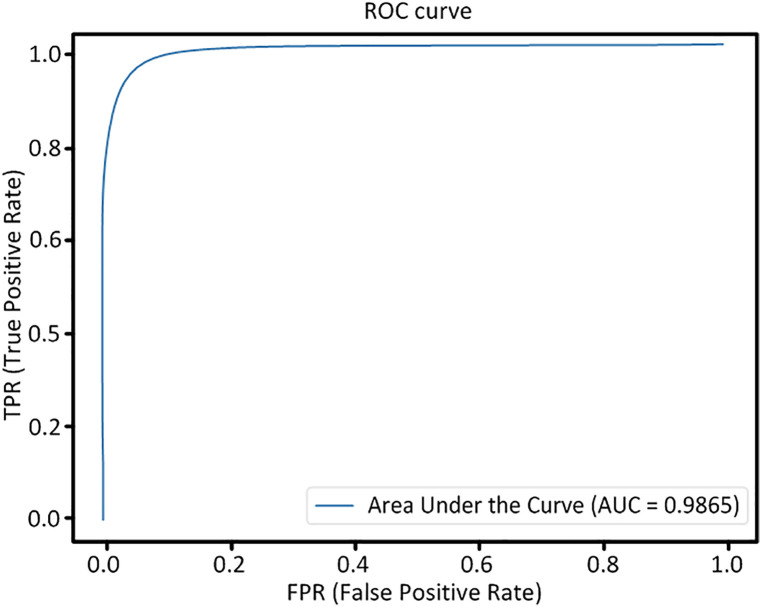
The ROC curve obtained from the CHASE DB1 dataset.

During the training process, the loss on the validation set serves as an effective indicator the training performance of the model. As illustrated in Fig 8, the model achieved optimal performance after approximately 150 iterations on both the DRIVE and CHASE DB1 datasets. The Receiver Operating Characteristic (ROC) curves depicted in Figs 9 and 10 offer a more comprehensive analysis the performance post-training of the model.

### Ablation experiment

In the baseline U-Net model, the integration of the HarDNet block results in a significant improvement in segmentation accuracy, increased by 0.93%. However, this enhancement is accompanied by a decrease in the F1 score. This phenomenon can potentially be attributed to the following: Although the dense connection structure in HarDNet improves the efficiency of feature reuse, it concurrently causes an over smoothing effect on certain fine grained features. Similarly, the incorporation of the RFB module alone leads to an increase in segmentation accuracy increased by 1%, but concurrently reduces both the Sp and F1 scores. This effect originates from the sparse sampling mechanism of dilated convolution. While expanding the coverage scope, it skips a substantial number of local points within the covered area. As a result, the local context information upon which the output eigenvalues depend becomes sparse and incomplete. Notably, when the HarDNet block and the RFB module are combined, there is a substantial improvement in the overall performance of the segmentation model, the accuracy rate increased by 1.6%, the Se rate increased by 6.7%. The details of these experiments are listed in Table 3.

**Table 3:** Ablation experiments on the DRIVE dataset.

Model	Se	Sp	F1	ACC
Baseline	0.7537	0.9820	0.8142	0.9531
Baseline+HarDNet	0.7623	0.9831	0.7970	0.9620
Baseline+RFB	0.7627	0.9817	0.8131	0.9631
Baseline+HarDNet + RFB	0.8042	0.9843	0.8178	0.9685

A comprehensive evaluation of the performance of the proposed network was conducted and compared with that of the traditional U-Net architecture. The assessment focused on parameter efficiency and overall effectiveness. Key metrics were analyzed, including the number of parameters, computational complexity (measured in FLOPs), time per iteration, and inference time per test data point. The results of this comparative analysis are summarized in Table 4.

**Table 4:** Comprehensive parameters comparative analysis of the proposed network with Unet.

Model	Parameters	Flops	Time (s/epoch)	Time (ms/img)
Unet	519202	1032931	200	350
Res-Unet	412593	846925	154	199
Proposed	364650	658237	45	167

## Discussion and conclusion

**Fig 11:**
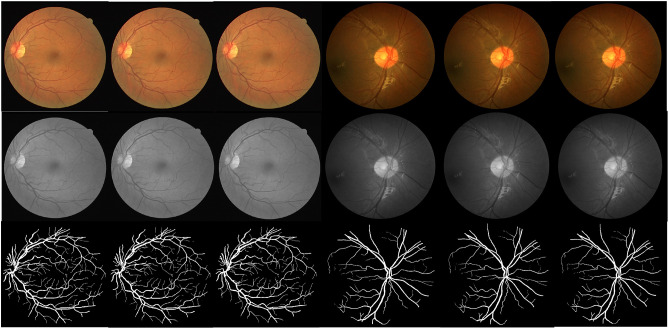
A comparison between the pre – processed images of two datasets and their corresponding gold standards is presented. The left – most three columns of images are derived from the DRIVE dataset, while the right – most three columns are from the CHASE DB1 dataset. In sequence from top to bottom are the original images, the pre – processed images, and the gold standards.

After the preprocessing of the original images, numerous fine blood vessels can be observed. However, these vessels are not depicted in the gold standard. This factor also constitutes a significant contributor to the final segmentation error (Fig 11).

The proposed method, which extends the HarDNet framework by incorporating Receive Field Block (RFB) and Dense Aggregation Block (DAB) modules, has shown superior performance on the DRIVE and CHASE_DB1 datasets. The DAB module effectively aggregates multi-scale features, enabling the model to capture fine-grained details critical for accurate segmentation. Additionally, the use of a composite loss function has further optimized training, resulting in improved segmentation accuracy. These architectural enhancements address some of the limitations of traditional methods, particularly in handling complex vascular structures and fine capillaries.

Despite these advancements, several challenges persist. First, the performance of the proposed model is highly sensitive to image quality. Low-quality images, often encountered in clinical settings due to factors such as patient movement, equipment limitations, or suboptimal lighting conditions, can lead to issues like vessel discontinuity and segmentation artifacts. Second, the limited size and inherent biases of existing datasets, including class imbalance between vascular and background pixels, pose significant challenges [27,35]. These biases can distort evaluation metrics and increase the risk of overfitting, limiting the generalizability of the model to unseen data. Third, dataset-specific variations, such as color contrast and domain shifts, further complicate the applicability of the model across diverse clinical environments [36,37]. In the final comparative experiment of the results, from the local magnification results, it can be seen that there are many phenomena of vascular discontinuity and breakage in the results due to the single-pixel appearance of blood vessels in the original image. These problems can be compensated for by increasing the data volume and post-processing techniques.

To address these limitations and enhance the clinical applicability of the proposed method, the following research directions was proposed:

1. Advanced Preprocessing and Noise-Robust Modules: Developing advanced preprocessing techniques or integrating noise-robust modules, such as denoising autoencoders or adversarial training methods, could improve the resilience of the model to low-quality or noisy images [ 38,39]. Preprocessing pipelines for image quality normalization and artifact removal should be explored to ensure consistent performance across diverse clinical environments.
2. Domain Adaptation and Generalization: Investigating domain adaptation techniques, such as domain adversarial training or style transfer, could mitigate dataset biases and enhance cross-dataset generalizability [40]. Cross-dataset validation should be implemented to assess the performance of the model on retinal images acquired under varying conditions and from diverse patient populations.
3. Multi-Modal Data Integration: Exploring the integration of multi-modal data, such as combining fundus images with optical coherence tomography (OCT) or other imaging modalities, could provide additional contextual information and improve segmentation accuracy. This approach would leverage complementary data from different imaging techniques, reducing the impact of dataset-specific biases.
4. Parameter Optimization and Experimental Enhancements: Future work should focus on deploying enhanced experimental platforms to facilitate comprehensive investigation of patch-based strategies and parameter optimization [41]. This would enable more robust tuning of the hyperparameters and improve its overall performance.
5. To address the issues of small sample size of data and the presence of single-pixel vessels in the original images, which cause vessel breakage in the segmentation results, data augmentation and post-processing techniques are employed to make up for these deficiencies.

Adaptive Preprocessing for Image Quality Variations: Developing adaptive preprocessing techniques and robust feature extraction methods to address image quality variations caused by lighting conditions, sensor noise, and compression artifacts is critical [42]. These techniques are expected to enhance adaptability to real-world clinical scenarios, as image quality can vary significantly in these situations.

In conclusion, the proposed method represents a significant step forward in retinal vessel segmentation, particularly in handling complex vascular structures and fine capillaries. However, challenges related to image quality, dataset biases, and generalizability remain. By pursuing the proposed research directions, we aim to enhance the robustness, generalizability, and clinical applicability of the model while ensuring its reliability in diverse real-world scenarios. These advancements will not only improve the accuracy of retinal vessel segmentation but also contribute to the broader field of medical image analysis, ultimately benefiting clinical diagnostics and patient care.
